# Supplementary material for: Providing Comprehensive Dietary Fatty Acid Profiling from Saturates to Polyunsaturates with the Malaysia Lipid Study-Food Frequency Questionnaire: Validation Using the Triads Approach
Source: Nutrients. 2020 Dec 31;13(1):120. doi: 10.3390/nu13010120 (PMC7823982; doi:10.3390/nu13010120)
Supplement: Supplementary file 1 [file nutrients-13-00120-s001.zip › nutrients-988928-Supplement Figures 1 and 2.docx]

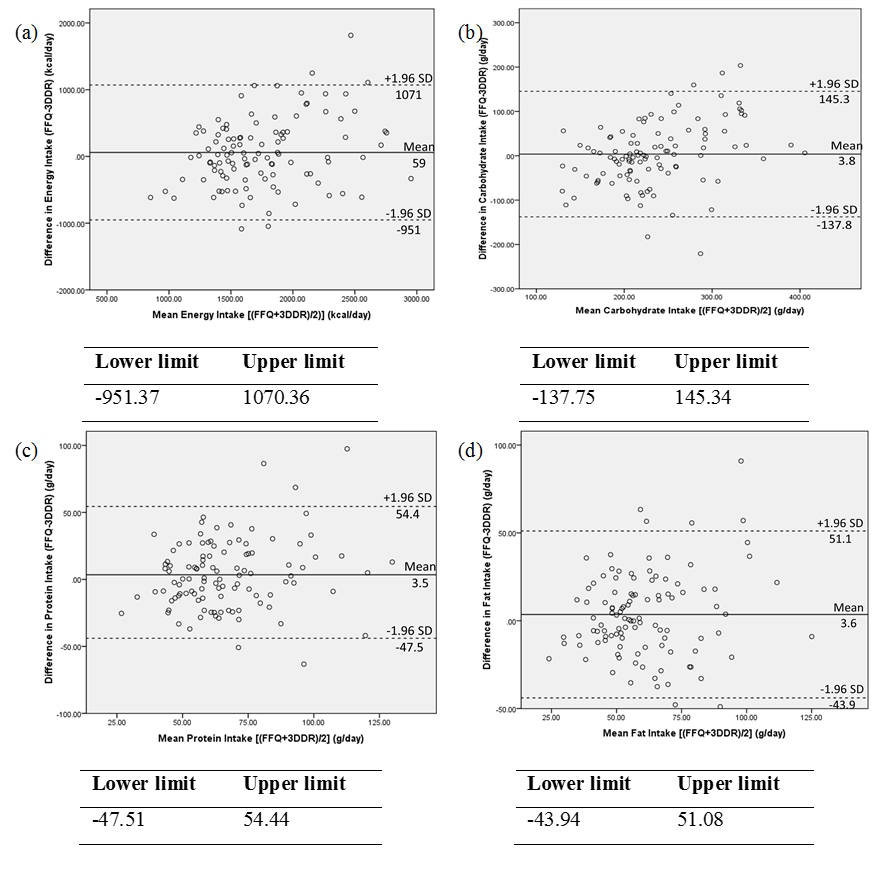


Abbreviations: DR=3-day dietary records; FFQ=food frequency questionnaires; g/day=gram per day.

**Figure S1** Bland-Altman Plots between FFQ and DR for (a) total energy (b) carbohydrate (c) protein (d) total fat


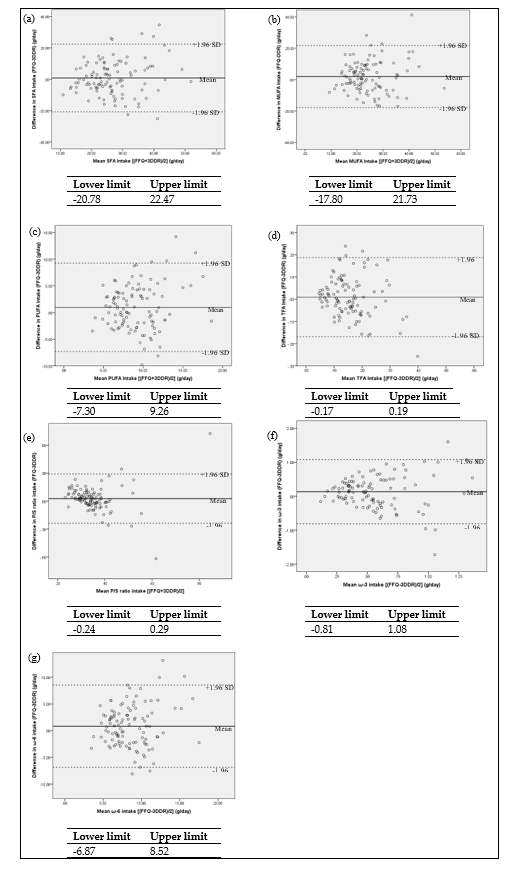


**Figure S2** Bland-Altman Plots between FFQ and DR for (a) SFA (b) MUFA (c) PUFA (d) TFA (e) P/S ratio (f) ω-3 FAs (g) ω-6 FAs; Abbreviations: DR=3-day dietary records; FFQ=food frequency questionnaires; MUFA=monounsaturated fatty acid; ω-3 FAs=omega-3 fatty acids; ω-6 FAs=omega-6 fatty acids; g/day=gram per day; PUFA=polyunsaturated fatty acid; P/S ratio= polyunsaturated fatty acid/saturated fatty acid ratio; SFA=saturated fatty acid; TFA=trans fatty acid.
