# Supplementary material for: Providing Comprehensive Dietary Fatty Acid Profiling from Saturates to Polyunsaturates with the Malaysia Lipid Study-Food Frequency Questionnaire: Validation Using the Triads Approach
Source: Nutrients. 2020 Dec 31;13(1):120. doi: 10.3390/nu13010120 (PMC7823982; doi:10.3390/nu13010120)
Supplement: Supplementary file 1 [file nutrients-13-00120-s001.zip › nutrients-988928-Supplement file 1-MLS FFQ Form.docx]

**Malaysia Lipid Study-FOOD FREQUENCY QUESTIONNAIRE**

**(MLS-FFQ)**

Code :

Q

**-**

Name :

Gender : Male / Female

Date :

**INFORMATION SHEET**

Thank you for agreeing to fill this questonnaire (FFQ) . The purpose of this FFQ is to assess your habitual food intake.

1. This FFQ has 10 (TEN) pages.
2. Please refer to page ii for guidance and methods in filling this questionnaire.
3. Our research staff are ready to assist you if you have queries about understanding the format of the FFQ.
4. You may also contact the researcher below for further queries:

Yeak Zu Wei

017-5707079

yeak_wei@hotmail.com

**GUIDELINES IN ANSWERING THIS FOOD FREQUENCY QUESTIONNAIRE (FFQ)**

1. This FFQ contains 240 food items to be answered.
2. You are required to check through all food items in the various categories given in the food list.
3. A food item that is not eaten in the past 3 months is considered as ‘not taken’.
4. If the food item is eaten, please tick (**🗸**) and answer the following row, if not, cross (**X**) the representative box.
5. You can refer to the examples below to answer the FFQ.

| **Column in the form** | **Descriptions** |
| --- | --- |
| **Food** | This column shows the list of foods that are potentially eaten by the subject |
| **Taken (YES/NO)** | This column is required to be filled as either tick (**🗸**) if eaten in the past 3 months or cross (**X**) if not. Any food item marked not taken (**X**) in the past 3 months, means no further information is required and you ,may proceed to the next food item.. |
| **Preparation method** | This column shows how the food is being prepared.  ***NOTE**: Stir-fried refers to *masak kicap, masak halia, tumis air, masak kunyit, kung pou, marmite, braise, stew* etc which is not batter-fried or deep-fried.  *Pre-fried*, *stir fried* refers to the food which is firstly deep fried followed by stir fried cooking method, e.gg *sweet sour, tiga rasa etc.* |
| **Frequency** | This column refers to how frequently the food item is being eaten per day, per week or per month. ‘Rare’ refers to the food which has been taken in the past 3 months but less than once a month. |
| **Household measurement unit** | There are several measurements which are used depending on the type of food.  **Piece** – describes intake of one particular single food item such buns, biscuits, *kuih* etc.  **Whole** – describes intake of the whole food item.  **Matchbox** – describes to edible portions of meat/fish products eaten as equivalent in size to a matchbox.  **Tablespoon/ Teaspoon** – describes to the measurement of food (eg. jam, sugar, coffee powder) using tablespoon/ teaspoon.  **Bowl** – describes the measurement of the food (eg. rice, noodles) using a bowl as per small, medium or large size.  **Glass/ Cup** – describes the measurement of beverages taken in a glass or cup.  **Serving** – describe the standard or normal portion of a set of food item, e.g. mee soup which contains yellow noodles, spring onion, meat and leafy vegetable. |
| **Portion taken** | This column shows the standard/normal quantity of food consumed by the subject in household measurement units. |
| **Extra gravy (1 tbsp)** | This column refers to extra gravy consumed from a food item in tablespoon measurement. |
| **Remarks** | Any special or additional information can be filled up in this column. |

***Abbreviation used in FFQ**

| **1.** | **Tbsp** | Tablespoon |
| --- | --- | --- |
| **2.** | **Tsp** | Teaspoon |
| **3.** | **S.bowl** | Small bowl |
| **4.** | **M.bowl** | Medium bowl |

**FOLLOW THE FLOW IN FILLING THE FFQ WITH THIS EXAMPLE:**

**Mr. A normally takes 2 pieces of *ayam masak merah* per week. For each piece, he realises that the edible portion is about 2 matchboxes. He also takes 2 tbsp of extra gravy for each piece of chicken every time. However, he doesn’t take any kind of beef.**

| **FOOD** | **Taken (YES/NO)** | **Preparation method** | **Frequency** | | | | **Household Measurement** | **Serving size** | **Extra gravy (tbsp)** | **Remarks** |
| --- | --- | --- | --- | --- | --- | --- | --- | --- | --- | --- |
|  |  |  | **Per day** | **Per week** | **Per month** | **Rarely** |  |  |  |  |
| Chicken | **🗸** | Masak merah |  | **2** |  |  | 1 matchbox | **2** | **2** |  |
| Beef | **X** | *Any* |  |  |  |  |  |  |  |  |

**4.** **Fill in** how many tablespoon(s) of extra gravy that is **consumed** together.

**3.** **Fill in** the **quantity** of food **eaten each time** by referring to the household measurement unit.

**2. Fill in** how frequently the food is eaten in **one day or week or month**.

**1. Tick 🗸 in the box** if the food is taken. **If not**, cross **X** in the box and continue with the next food item.

**5. Cross ‘X’ in the box** if the food is taken and continue with the next food item.

| **No.** | **FOOD** | **Taken (YES/ NO)** | **Preparation method** | **Frequency** | | | | **Household Measurement** | **Portion taken** | **Extra gravy (1 tbsp)** | **Remarks** |
| --- | --- | --- | --- | --- | --- | --- | --- | --- | --- | --- | --- |
|  |  |  |  | **per day** | **per week** | **per month** | **rarely** |  |  |  |  |
| **Cereals & Products** | |  |  |  |  |  |  |  |  |  |  |
| **A** | **Biscuits** | | | | | | | | | | |
| 1 | Milk/ Marie |  |  |  |  |  |  | 1 piece |  |  |  |
| 2 | Sandwich/ Jam/ Cream |  |  |  |  |  |  | 1 piece |  |  |  |
| 3 | Butter |  |  |  |  |  |  | 1 piece |  |  |  |
| 4 | Cream cracker |  |  |  |  |  |  | 1 piece |  |  |  |
| 5 | Fiber/ Wholemeal/ Multigrain |  |  |  |  |  |  | 1 piece |  |  |  |
| **B** | **Bread** | | | | | | | | | | |
| 6 | White |  |  |  |  |  |  | 1 slice |  |  |  |
| 7 | Fiber/ Wholemeal/ Multigrain |  |  |  |  |  |  | 1 slice |  |  |  |
| 8 | Flavoured/ Fruit |  |  |  |  |  |  | 1 slice |  |  |  |
| **C** | **Bun** | | | | | | | | | | |
| 9 | Plain/ flavoured |  |  |  |  |  |  | 1 piece |  |  |  |
| 10 | Sweeting filling (coconut, red beans, etc) |  |  |  |  |  |  | 1 piece |  |  |  |
| 11 | Savoury filling (ikan bilis, chicken, etc) |  |  |  |  |  |  | 1 piece |  |  |  |
| **D** | **Others * please specify the amount of dhal/ curry if taken** | | | | | | | | | | |
| 12 | Chapati* |  |  |  |  |  |  | 1 piece |  |  |  |
| 13 | Roti canai (Plain)* |  |  |  |  |  |  | 1 piece |  |  |  |
| 14 | Roti canai (Telur)* |  |  |  |  |  |  | 1 piece |  |  |  |
| 15 | Thosai* |  |  |  |  |  |  | 1 piece |  |  |  |
| 16 | Idli* |  |  |  |  |  |  | 1 piece |  |  |  |
| 17 | Uppuma |  |  |  |  |  |  | 1 piece |  |  |  |
| 18 | Roti naan* |  |  |  |  |  |  | 1 piece |  |  |  |
| 19 | Breakfast cereal (only) |  |  |  |  |  |  | 1 cup |  |  |  |
| 20 | Oats (uncooked) |  |  |  |  |  |  | 1 tbsp |  |  |  |
| **Cooked Rice** | | | | | | | | | | | |
| 21 | White rice |  |  |  |  |  |  | 1 s.bowl |  |  |  |
| 22 | Brown rice |  |  |  |  |  |  | 1 s.bowl |  |  |  |
| 23 | Porridge |  |  |  |  |  |  | 1 s.bowl |  |  |  |
| 24 | Nasi minyak |  |  |  |  |  |  | 1 s.bowl |  |  |  |
| 25 | Glutinous rice |  |  |  |  |  |  | 1 s.bowl |  |  |  |
| 26 | Tamarind rice |  |  |  |  |  |  | 1 s.bowl |  |  |  |
| 27 | Fried rice (standard set) |  |  |  |  |  |  | 1 serving |  |  |  |
| 28 | Briyani rice (Chicken/ Mutton/ Fish) |  |  |  |  |  |  | 1 serving |  |  |  |
| 29 | Chicken rice (Chicken/ Roasted pork/ Char siew) |  |  |  |  |  |  | 1 serving |  |  |  |
| 30 | Nasi lemak (standard set) |  |  |  |  |  |  | 1 serving |  |  |  |
| 31 | Nasi Tomato (standard set) |  |  |  |  |  |  | 1 serving |  |  |  |
| **Noodles** | | | | | | | | | | | |
| **A** | **Soup- based noodles (including yellow noodles, meehoon, kuey teow)** | | | | | | | | | | |
| 32 | Noodles with clear soup (eg. Mee soup) |  |  |  |  |  |  | 1 serving |  |  |  |
| 33 | Asam laksa |  |  |  |  |  |  | 1 serving |  |  |  |
| 34 | Curry laksa |  |  |  |  |  |  | 1 serving |  |  |  |
| 35 | Ladna/ Kung fu |  |  |  |  |  |  | 1 serving |  |  |  |
| 36 | Bandung |  |  |  |  |  |  | 1 serving |  |  |  |
| 37 | Jawa/ Rebus |  |  |  |  |  |  | 1 serving |  |  |  |
| 38 | Wan tan |  |  |  |  |  |  | 1 serving |  |  |  |
| 39 | Prawn |  |  |  |  |  |  | 1 serving |  |  |  |
| **B** | **Non- soup based noodles (including yellow noodles, meehoon, kuey teow)** | | | | | | | | | | |
| 40 | Char Kuey Teow |  |  |  |  |  |  | 1 serving |  |  |  |
| 41 | Fried (economic) |  |  |  |  |  |  | 1 m.bowl |  |  |  |
| 42 | Hokkien mee/ Braised |  |  |  |  |  |  | 1 m.bowl |  |  |  |
| 43 | Kicap/ kolok |  |  |  |  |  |  | 1 m.bowl |  |  |  |
| 44 | Wan tan |  |  |  |  |  |  | 1 m.bowl |  |  |  |
| **C** | **Pasta [Spaghetti/Fettucini etc]** | | | | | | | | | | |
| 45 | Tomato sauce |  |  |  |  |  |  | 1 serving |  |  |  |
| 46 | Cheese/ Meat sauce |  |  |  |  |  |  | 1 serving |  |  |  |
| **D** | **Others** | | | | | | | | | | |
| 47 | Chee Cheong Fun |  |  |  |  |  |  | 1 s.bowl |  |  |  |
| 48 | Instant noodles |  |  |  |  |  |  | 1 packet |  |  |  |
| **Traditional Kuih** | | | | | | | | | | | |
| **A** | **Malay Kuih *please specify the amount of curry or chilli sauce used** | | | | | | | | | | |
| 49 | Cekodok |  |  |  |  |  |  | 1 piece |  |  |  |
| 50 | Cucur badak |  |  |  |  |  |  | 1 piece |  |  |  |
| 51 | Cucur (sayur/ udang/ ikan bilis) |  |  |  |  |  |  | 1 piece |  |  |  |
| 52 | Roti jala |  |  |  |  |  |  | 1 piece |  |  |  |
| 53 | Apam balik |  |  |  |  |  |  | 1 piece |  |  |  |
| 54 | Gandum+ Santan *Kuih bakar, kuih ketayap, bahulu* |  |  |  |  |  |  | 1 piece |  |  |  |
| 55 | Keropok lekor * |  |  |  |  |  |  | 1 piece |  |  |  |
| 56 | Kuih Kasturi |  |  |  |  |  |  | 1 piece |  |  |  |
| 57 | Pisang/ Cempedak/ Sukun/ Keledek goreng |  |  |  |  |  |  | 1 piece |  |  |  |
| 58 | Kuih pulut pangang |  |  |  |  |  |  | 1 piece |  |  |  |
| 59 | Kuih bom |  |  |  |  |  |  | 1 piece |  |  |  |
| 60 | Pulut+Santan *Kuih badak berendam, kuih kasui, seri muka, kuih koci, kuih pulut* |  |  |  |  |  |  | 1 piece |  |  |  |
| 61 | Rempeyek |  |  |  |  |  |  | 1 piece |  |  |  |
| 62 | Kuih bingka |  |  |  |  |  |  | 1 piece |  |  |  |
| 63 | Rice-santan *Kuih bungkus, kuih lompang, kuih pelita, lepat pisang, onde-onde, pulut bulu/putu piring. Kuih apam* |  |  |  |  |  |  | 1 piece |  |  |  |
| 64 | Kuih keria |  |  |  |  |  |  | 1 piece |  |  |  |
| 65 | Sago/ Tapioca + Santan *Kuih bangkit, kuih kastard, kuih lapis, kuih lepat ubi, kuih sago, kuih talam ubi* |  |  |  |  |  |  | 1 piece |  |  |  |
| **B** | **Chinese Kuih * please specify the amount of curry or chilli sauce used** | | | | | | | | | | |
| 66 | Nyonya kuih - with filling  *(Ang ku kueh, Loh mai chee)* |  | Steamed |  |  |  |  | 1 piece |  |  |  |
| 67 | Nyonya kuih w/out filling  *(Huat Kueh, egg pancake)* |  | Steamed |  |  |  |  | 1 piece |  |  |  |
| 68 | Bak zhang, pork *(chinese rice dumpling)* |  | Steamed |  |  |  |  | 1 piece |  |  |  |
| 69 | Ki zhang, red/ mung bean  *(chinese rice dumpling)* |  | Steamed |  |  |  |  | 1 piece |  |  |  |
| 70 | Deep-fried kuih - *(Ham chi peng/Chinese pancake)* |  |  |  |  |  |  | 1 piece |  |  |  |
| 71 | White radish/ Yam cake * |  |  |  |  |  |  | 1 piece |  |  |  |
| 72 | Yau Char Kway |  | Deep-Fried |  |  |  |  | 1 piece |  |  |  |
| 73 | Loh mai kai/ Lotus leaf rice/ Yam rice * |  |  |  |  |  |  | 1 piece |  |  |  |
| 74 | Pao (pork, char siew) * |  | Steamed |  |  |  |  | 1 piece |  |  |  |
| 75 | Pau (kaya, kelapa) |  | Steamed |  |  |  |  | 1 piece |  |  |  |
| 76 | Pau (red bean, lotus seed) |  | Steamed |  |  |  |  | 1 piece |  |  |  |
| 77 | Pau (chicken curry) |  | Steamed |  |  |  |  | 1 piece |  |  |  |
| 78 | Steam bun (kikaku, mantou) |  | Steamed |  |  |  |  | 1 piece |  |  |  |
| **C** | **Indian Kuih** | | | | | | | | | | |
| 79 | Kesari |  |  |  |  |  |  | 1 piece |  |  |  |
| 80 | Laddu |  |  |  |  |  |  | 1 piece |  |  |  |
| 81 | Muruku |  |  |  |  |  |  | 1 s.bowl |  |  |  |
| 82 | Paal appam |  |  |  |  |  |  | 1 piece |  |  |  |
| 83 | Palkova |  |  |  |  |  |  | 1 piece |  |  |  |
| 84 | Payasam |  |  |  |  |  |  | 1 s.bowl |  |  |  |
| 85 | Ponggal rice |  |  |  |  |  |  | 1 s.bowl |  |  |  |
| 86 | Putu mayam (grated coconut/ sugar) |  |  |  |  |  |  | 1 s.bowl |  |  |  |
| 87 | Vadai (dhal/ulunthu) |  |  |  |  |  |  | 1 piece |  |  |  |
| **Desserts, Snacks & Confectionary** | | | | | | | | | | | |
| 88 | Butter cake |  |  |  |  |  |  | 1 slice |  |  |  |
| 89 | Cheese cake |  |  |  |  |  |  | 1 slice |  |  |  |
| 90 | Cream cake |  |  |  |  |  |  | 1 slice |  |  |  |
| 91 | Sponge cake |  |  |  |  |  |  | 1 slice |  |  |  |
| 92 | Cream puff |  |  |  |  |  |  | 1 piece |  |  |  |
| 93 | Chocolate |  |  |  |  |  |  | 1 matchbox |  |  |  |
| 94 | Kerepek bawang/ kentang/ ubi/ tepung/ plantain |  |  |  |  |  |  | 1 s.bowl |  |  |  |
| 95 | Keropok ikan/ udang |  |  |  |  |  |  | 1 s.bowl |  |  |  |
| 96 | Popcorn |  |  |  |  |  |  | 1 s.bowl |  |  |  |
| 97 | Snack food (eg. Mamee, Super ring, Double Decker etc) |  |  |  |  |  |  | 1 packet |  |  |  |
| 98 | Waffle |  |  |  |  |  |  | 1 set |  |  |  |
| 99 | Egg tart |  |  |  |  |  |  | 1 piece |  |  |  |
| 100 | Currypuff (potato, sardine, chicken) |  |  |  |  |  |  | 1 piece |  |  |  |
| 101 | Donut (with filling/ topping) |  |  |  |  |  |  | 1 piece |  |  |  |
| 102 | Jelly |  |  |  |  |  |  | 1 piece |  |  |  |
| 103 | Muffin |  |  |  |  |  |  | 1 piece |  |  |  |
| 104 | Local soup dessert, with santan (*Bubur chacha, bubur jagung, bubur kacang hijau/ merah, pulut hitam etc)* |  |  |  |  |  |  | 1 m.bowl |  |  |  |
| 105 | Ais Kacang |  |  |  |  |  |  | 1 m.bowl |  |  |  |
| 106 | Cendol with/ without pulut |  |  |  |  |  |  | 1 m.bowl |  |  |  |
| 107 | Local soup dessert, without santan (*Lai Chi Kang, lotus seed, sea coconut, sea bird nest, bird nest, red date etc)* |  |  |  |  |  |  | 1 m.bowl |  |  |  |
| **Meat, Poultry & Products** | | | | | | | | | | | |
| 108 | Beef   *Please specify the amount of gravy |  | Curry without santan |  |  |  |  | 1 matchbox |  |  | Plain/ Evaporated milk/ Low fat milk |
|  |  |  | Curry/ rendang/ kurma/ lemak |  |  |  |  | 1 matchbox |  |  |  |
|  |  |  | Deep-fried |  |  |  |  | 1 matchbox |  |  |  |
|  |  |  | Grilled/ pan-fried |  |  |  |  | 1 matchbox |  |  |  |
|  |  |  | Sambal/ masak merah |  |  |  |  | 1 matchbox |  |  |  |
|  |  |  | Soup |  |  |  |  | 1 matchbox |  |  |  |
|  |  |  | Stir fried |  |  |  |  | 1 matchbox |  |  |  |
| 109 | Mutton/ lamb  *Please specify the amount of gravy |  | Curry without santan |  |  |  |  | 1 matchbox |  |  | Plain/ Evaporated milk/ Low fat milk |
|  |  |  | Peratal/ masala/ varuval/ curry/ rendang |  |  |  |  | 1 matchbox |  |  |  |
|  |  |  | Stir fried |  |  |  |  | 1 matchbox |  |  |  |
|  |  |  | Steam/soup |  |  |  |  | 1 matchbox |  |  |  |
|  |  |  | Chops/ Grilled/ pan-fried |  |  |  |  | 1 matchbox |  |  |  |
| 110 | Chicken  *Please specify the amount of gravy  *Deep-fried chicken does not include food from fast food chain, e.g. fried chicken from KFC, McDonald's etc* |  | Curry without santan |  |  |  |  | 1 matchbox |  |  | Plain/ Evaporated milk/ Low fat milk |
|  |  |  | Masala/ peratal/ varuval/ curry |  |  |  |  | 1 matchbox |  |  |  |
|  |  |  | Rendang/ lemak |  |  |  |  | 1 matchbox |  |  |  |
|  |  |  | Batter-fried/ chops |  |  |  |  | 1 matchbox |  |  |  |
|  |  |  | Deep-fried |  |  |  |  | 1 matchbox |  |  |  |
|  |  |  | Grilled/ pan-fried |  |  |  |  | 1 matchbox |  |  |  |
|  |  |  | Roasted |  |  |  |  | 1 matchbox |  |  |  |
|  |  |  | Sambal/ masak merah |  |  |  |  | 1 matchbox |  |  |  |
|  |  |  | Pre-fried, stir fried |  |  |  |  | 1 matchbox |  |  |  |
|  |  |  | Stir fried |  |  |  |  | 1 matchbox |  |  |  |
|  |  |  | Steam/ soup |  |  |  |  | 1 matchbox |  |  |  |
| 111 | Chicken Sausage/ Nugget |  | Pan/ Deep-fried |  |  |  |  | 1 piece |  |  |  |
| 112 | Pork*Please specify the amount of gravy |  | Curry |  |  |  |  | 1 matchbox |  |  |  |
|  |  |  | Deep-fried |  |  |  |  | 1 matchbox |  |  |  |
|  |  |  | Grilled/ pan-fried |  |  |  |  | 1 matchbox |  |  |  |
|  |  |  | Stir fried |  |  |  |  | 1 matchbox |  |  |  |
|  |  |  | Pre-fried, stir fried |  |  |  |  | 1 matchbox |  |  |  |
|  |  |  | Roasted |  |  |  |  | 1 matchbox |  |  |  |
|  |  |  | Char siew |  |  |  |  | 1 matchbox |  |  |  |
| 113 | Pork Sausage/ Ham/ Bacon |  | Pan/ Deep-fried |  |  |  |  | 1 matchbox |  |  |  |
| 114 | Duck |  | Roasted |  |  |  |  | 1 matchbox |  |  |  |
| 115 | Satay * Specify the amount of *kuah kacang* |  |  |  |  |  |  |  |  |  |  |
|  | Beef |  |  |  |  |  |  | 1 stick |  |  |  |
|  | Chicken |  |  |  |  |  |  | 1 stick |  |  |  |
|  | Mutton |  |  |  |  |  |  | 1 stick |  |  |  |
| 116 | Meatball | | | | | | | | | | |
|  | Beef |  |  |  |  |  |  | 1 piece |  |  |  |
|  | Chicken |  |  |  |  |  |  | 1 piece |  |  |  |
|  | Pork |  |  |  |  |  |  | 1 piece |  |  |  |
| 117 | Eggs *including chicken egg and duck egg* *specify the amount of gravy if served with curry, sambal or sweet & sour sauce |  | Boiled/ steamed |  |  |  |  | 1 whole |  |  |  |
|  |  |  | Omelette/ Scrambled |  |  |  |  | 1 whole |  |  |  |
|  |  |  | Fried |  |  |  |  | 1 whole |  |  |  |
|  |  |  | Salted |  |  |  |  | 1 whole |  |  |  |
| **Internal Organs** | | | | | | | | | | | |
| 118 | Beef internal organ |  | Masak lemak |  |  |  |  | 1 matchbox |  |  |  |
|  |  |  | Masak merah |  |  |  |  | 1 matchbox |  |  |  |
|  |  |  | Deep fried |  |  |  |  | 1 matchbox |  |  |  |
| 119 | Chicken internal organ |  | deep fried |  |  |  |  | 1 matchbox |  |  |  |
| 120 | Pork internal organ |  | Soup |  |  |  |  | 1 matchbox |  |  |  |
|  |  |  | Stir fried |  |  |  |  | 1 matchbox |  |  |  |
| **Fish, Shellfish & products** | | | | | | | | | | | |
| 121 | Anchovies |  | Deep fried |  |  |  |  | 1 tbsp |  |  |  |
|  |  |  | Sambal |  |  |  |  | 1 tbsp |  |  |  |
| 122 | Low fat fishes   kikek  bulus-bulus  toman  stingray  grouper  seluang  nyior-nyior  baung   **Specify the amount of gravy if served with curry, sambal or sweer & sour sauce* |  | Asam pedas |  |  |  |  | 1 matchbox |  |  |  |
|  |  |  | Curry |  |  |  |  | 1 matchbox |  |  |  |
|  |  |  | Deep fried* |  |  |  |  | 1 matchbox |  |  |  |
|  |  |  | Grilled |  |  |  |  | 1 matchbox |  |  |  |
|  |  |  | Steam/soup |  |  |  |  | 1 matchbox |  |  |  |
| 123 | Moderate fat fishes  dory  cencaru  black  pomfret   sepat  ikan parang  tenggiri  gelama  jenahak  pelata  kerisi  merah  selar  tilapia  belida   **Specify the amount of gravy if served with curry, sambal or sweer & sour sauce* |  | Asam pedas |  |  |  |  | 1 matchbox |  |  |  |
|  |  |  | Batter fried* |  |  |  |  | 1 matchbox |  |  |  |
|  |  |  | Curry |  |  |  |  | 1 matchbox |  |  |  |
|  |  |  | Deep fried* |  |  |  |  | 1 matchbox |  |  |  |
|  |  |  | Grilled |  |  |  |  | 1 matchbox |  |  |  |
|  |  |  | Lemak |  |  |  |  | 1 matchbox |  |  |  |
|  |  |  | Sambal/ merah |  |  |  |  | 1 matchbox |  |  |  |
|  |  |  | Steam/ soup |  |  |  |  | 1 matchbox |  |  |  |
|  |  |  | Stir fried |  |  |  |  | 1 matchbox |  |  |  |
|  |  |  | Pre-fried, stir fried |  |  |  |  | 1 matchbox |  |  |  |
|  |  |  |  |  |  |  |  |  |  |  |  |
| 124 | High fat fishes   siakap  keli  patin  senangin  kembong  white  pomfret   **Specify the amount of gravy if served with curry, sambal or sweer & sour sauce* |  | Asam pedas |  |  |  |  | 1 matchbox |  |  |  |
|  |  |  | Curry |  |  |  |  | 1 matchbox |  |  |  |
|  |  |  | Deep fried* |  |  |  |  | 1 matchbox |  |  |  |
|  |  |  | Grilled |  |  |  |  | 1 matchbox |  |  |  |
|  |  |  | Lemak |  |  |  |  | 1 matchbox |  |  |  |
|  |  |  | Sambal/ merah |  |  |  |  | 1 matchbox |  |  |  |
|  |  |  | Steam/ soup |  |  |  |  | 1 matchbox |  |  |  |
|  |  |  | Stir fried |  |  |  |  | 1 matchbox |  |  |  |
|  |  |  | Pre-fried, stir fried |  |  |  |  | 1 matchbox |  |  |  |
| 125 | Deep sea cold fish   tuna  salmon  sardine  codfish |  | Canned |  |  |  |  | 1 matchbox |  |  |  |
|  |  |  | Curry |  |  |  |  | 1 matchbox |  |  |  |
|  |  |  | Deep fried |  |  |  |  | 1 matchbox |  |  |  |
|  |  |  | Grilled |  |  |  |  | 1 matchbox |  |  |  |
|  |  |  | Lemak |  |  |  |  | 1 matchbox |  |  |  |
|  |  |  | Raw |  |  |  |  | 1 matchbox |  |  |  |
|  |  |  | Sambal |  |  |  |  | 1 matchbox |  |  |  |
|  |  |  | Steam/soup |  |  |  |  | 1 matchbox |  |  |  |
| 126 | Prawn/ Shrimp  **Specify the amount of gravy if served with curry, sambal or sweer & sour sauce* |  | Butter |  |  |  |  | 1 piece |  |  |  |
|  |  |  | curry/masala |  |  |  |  | 1 piece |  |  |  |
|  |  |  | Batter fried* |  |  |  |  | 1 piece |  |  |  |
|  |  |  | Deep fried |  |  |  |  | 1 piece |  |  |  |
|  |  |  | Lemak |  |  |  |  | 1 piece |  |  |  |
|  |  |  | sambal |  |  |  |  | 1 piece |  |  |  |
|  |  |  | soup |  |  |  |  | 1 piece |  |  |  |
|  |  |  | Stir fried |  |  |  |  | 1 piece |  |  |  |
|  |  |  | Pre-fried, stir fried |  |  |  |  | 1 piece |  |  |  |
| 127 | Shell fish  ( served without shell) (eg. Cockles, kepah, lala, etc) |  | Curry/ rendang |  |  |  |  | 1 s.bowl |  |  |  |
|  |  |  | Lemak |  |  |  |  | 1 s.bowl |  |  |  |
|  |  |  | Sambal |  |  |  |  | 1 s.bowl |  |  |  |
|  |  |  | Soup/boiled |  |  |  |  | 1 s.bowl |  |  |  |
|  |  |  | Stir fried |  |  |  |  | 1 s.bowl |  |  |  |
| 128 | Squid,cuttlefish, jelly fish  **Specify the amount of gravy if served with curry, sambal or sweer & sour sauce* |  | Batter fried* |  |  |  |  | 1 matchbox |  |  |  |
|  |  |  | grilled |  |  |  |  | 1 matchbox |  |  |  |
|  |  |  | sambal |  |  |  |  | 1 matchbox |  |  |  |
|  |  |  | Soup/ boiled |  |  |  |  | 1 matchbox |  |  |  |
|  |  |  | Stir fried |  |  |  |  | 1 matchbox |  |  |  |
| 129 | Processed food/ Frozen food *e.g. fish ball, fish cake, crabstick, prawn ball, squid ball, etc.* |  | Deep fried/ stir fried |  |  |  |  | 1 piece |  |  |  |
|  |  |  | Steam/ soup |  |  |  |  | 1 piece |  |  |  |
| 130 | Otak-otak |  |  |  |  |  |  | 1 matchbox |  |  |  |
| 131 | Salted fish |  | Fried |  |  |  |  | 1 matchbox |  |  |  |
| **Legumes** | | | | | | | | | | | |
| 132 | Legumes (baked bean, black bean, chickpea, dhal, kidney bean etc) *not including dhal from roti canai, naan, thosai. |  | stir-fried |  |  |  |  | 1 s.bowl |  |  |  |
|  |  |  | boiled/ steam/ soup |  |  |  |  | 1 s.bowl |  |  |  |
|  |  |  | curry |  |  |  |  | 1 s.bowl |  |  |  |
|  |  |  | deep fried |  |  |  |  | 1 s.bowl |  |  |  |
| 133 | Tempeh |  | deep fried |  |  |  |  | 1 s.bowl |  |  |  |
|  |  |  | stir-fried |  |  |  |  | 1 s.bowl |  |  |  |
|  |  |  | Sambal |  |  |  |  | 1 s.bowl |  |  |  |
| 134 | Fucuk/ Food analog |  | stir fried |  |  |  |  | 1 s.bowl |  |  |  |
|  |  |  | deep fried |  |  |  |  | 1 s.bowl |  |  |  |
|  |  |  | Soup |  |  |  |  | 1 s.bowl |  |  |  |
| 135 | Tauhoo/ Taukua/ Egg tofu |  | deep fried |  |  |  |  | 1 whole |  |  |  |
|  |  |  | stir fried |  |  |  |  | 1 whole |  |  |  |
|  |  |  | Soup/ steam |  |  |  |  | 1 whole |  |  |  |
|  |  |  | Curry |  |  |  |  | 1 whole |  |  |  |
|  |  |  | Sambal |  |  |  |  | 1 whole |  |  |  |
| 136 | Tauhoo pok |  | Curry |  |  |  |  | 1 whole |  |  |  |
|  |  |  | stir fried |  |  |  |  | 1 whole |  |  |  |
|  |  |  | Soup |  |  |  |  | 1 whole |  |  |  |
| 137 | Peanut |  | Steam/ Boil |  |  |  |  | 1 s.bowl |  |  |  |
|  |  |  | Roasted |  |  |  |  | 1 s.bowl |  |  |  |
|  |  |  | Oil-roasted |  |  |  |  | 1 s.bowl |  |  |  |
|  |  |  | Deep fried, coated |  |  |  |  | 1 s.bowl |  |  |  |
| 138 | Papadam |  |  |  |  |  |  | 1 piece |  |  |  |
| 139 | Tau Foo Fah |  |  |  |  |  |  | 1 s.bowl |  |  |  |
| 140 | Cashew nut |  |  |  |  |  |  | 1 s.bowl |  |  |  |
| 141 | Pumpkin seed |  |  |  |  |  |  | 1 s.bowl |  |  |  |
| 142 | Sunflower seed |  |  |  |  |  |  | 1 s.bowl |  |  |  |
| 143 | Walnut |  |  |  |  |  |  | 1 s.bowl |  |  |  |
| **Vegetables** | | | | | | | | | | | |
| 144 | Brinjal |  | Stir fried |  |  |  |  | 1 s.bowl |  |  |  |
|  |  |  | Curry |  |  |  |  | 1 s.bowl |  |  |  |
|  |  |  | Steam/ soup |  |  |  |  | 1 s.bowl |  |  |  |
| 145 | Mixed vegetable (all green leafy vegetables, carrot, cauliflower, broccoli, cabbage, long beans, mushrooms, pickled vegetables, salted vegetables, etc) |  | Curry |  |  |  |  | 1 s.bowl |  |  |  |
|  |  |  | Stir-fried |  |  |  |  | 1 s.bowl |  |  |  |
|  |  |  | Soup |  |  |  |  | 1 s.bowl |  |  |  |
|  |  |  | Sambal belacan |  |  |  |  | 1 s.bowl |  |  |  |
|  |  |  | Masak lemak |  |  |  |  | 1 s.bowl |  |  |  |
|  |  |  | Acar |  |  |  |  | 1 s.bowl |  |  |  |
|  |  |  | Deep-fried |  |  |  |  | 1 s.bowl |  |  |  |
| 146 | Tempura |  | Batter-fried |  |  |  |  | 1 s.bowl |  |  |  |
| 147 | Starchy vegetable (potato, sweet potato, yam, tapioca, corn, pumpkin, etc) |  | Steam/ soup |  |  |  |  | 1 s.bowl |  |  |  |
|  |  |  | Stir-fried |  |  |  |  | 1 s.bowl |  |  |  |
|  |  |  | Curry |  |  |  |  | 1 s.bowl |  |  |  |
|  |  |  | Masak lemak |  |  |  |  | 1 s.bowl |  |  |  |
| **Fruits & Products** | | | | | | | | | | | |
| 148 | Apple |  |  |  |  |  |  | 1 whole |  |  |  |
| 149 | Avocado |  |  |  |  |  |  | 1 whole |  |  |  |
| 150 | Banana |  |  |  |  |  |  | 1 whole |  |  |  |
| 151 | Durian |  |  |  |  |  |  | 1 piece |  |  |  |
| 152 | Grapes |  |  |  |  |  |  | 1 piece |  |  |  |
| 153 | Guava |  |  |  |  |  |  | 1 slice |  |  |  |
| 154 | Honeydew |  |  |  |  |  |  | 1 slice |  |  |  |
| 155 | Longan/Lychee |  |  |  |  |  |  | 1 piece |  |  |  |
| 156 | Mango |  |  |  |  |  |  | 1 whole |  |  |  |
| 157 | Orange/ mandarin orange |  |  |  |  |  |  | 1 whole |  |  |  |
| 158 | Papaya |  |  |  |  |  |  | 1 slice |  |  |  |
| 159 | Pear/ Lai |  |  |  |  |  |  | 1 whole |  |  |  |
| 160 | Persimmon |  |  |  |  |  |  | 1 whole |  |  |  |
| 161 | Pineapple |  |  |  |  |  |  | 1 slice |  |  |  |
| 162 | Rambutan |  |  |  |  |  |  | 1 piece |  |  |  |
| 163 | Raisin |  |  |  |  |  |  | 1 tbsp |  |  |  |
| 164 | Pickled/ Dried fruit |  |  |  |  |  |  | 1 cup |  |  |  |
| 165 | Watermelon |  |  |  |  |  |  | 1 slice |  |  |  |
| **Milk & Dairy Products** | | | | | | | | | | | |
| 166 | Ice cream, milk based |  |  |  |  |  |  | 1 stick/ scoop |  |  |  |
| 167 | UHT milk (Full cream/ Low Fat/ Skimmed) |  |  |  |  |  |  | 1 glass |  |  |  |
| 168 | Milk powder (Full cream/ Low Fat/ Skimmed) |  |  |  |  |  |  | 1 tbsp |  |  |  |
| 169 | Yogurt (Full fat/ Low fat/ Skimmed) |  |  |  |  |  |  | 1 cup |  |  |  |
| 170 | Yogurt drink/ Lassi |  |  |  |  |  |  | 1 glass |  |  |  |
| 171 | Cheese |  |  |  |  |  |  | 1 slice |  |  |  |
| 172 | Creamer, non-dairy |  |  |  |  |  |  | 1 tsp |  |  |  |
| **Jam & Spread** | | | | | | | | | | | |
| 173 | Butter |  |  |  |  |  |  | 1 tsp |  |  |  |
| 174 | Chocolate spread |  |  |  |  |  |  | 1 tsp |  |  |  |
| 175 | Fruit Jam |  |  |  |  |  |  | 1 tsp |  |  |  |
| 176 | Kaya |  |  |  |  |  |  | 1 tsp |  |  |  |
| 177 | Margarine |  |  |  |  |  |  | 1 tsp |  |  |  |
| 178 | Peanut butter |  |  |  |  |  |  | 1 tsp |  |  |  |
| 179 | Honey |  |  |  |  |  |  | 1 tbsp |  |  |  |
| **Beverages** | | | | | | | | | | | |
| **A** | **Alcoholic beverages** | | | | | | | | | | |
| 180 | Beer |  |  |  |  |  |  | 1 glass |  |  |  |
| 181 | Wine |  |  |  |  |  |  | 1 glass |  |  |  |
| 182 | Whiskey |  |  |  |  |  |  | 1 glass |  |  |  |
| **B** | **Coffee/ Tea** | | | | | | | | | | |
| 183 | Condensed/ Evaporated/ RTD |  |  |  |  |  |  | 1 glass |  |  |  |
| 184 | O |  |  |  |  |  |  | 1 glass |  |  |  |
| 185 | Fresh milk/ UHT milk |  |  |  |  |  |  | 1 glass |  |  |  |
| 186 | 3 in 1 |  |  |  |  |  |  | 1 sachet |  |  |  |
| 187 | Ice blended/ Brewed (e.g. cappucino, mocha,machiato) |  |  |  |  |  |  | 1 cup |  |  |  |
| **C** | **Malted/ Cereal/ Chocolate drink** | | | | | | | | | | |
| 188 | Plain |  |  |  |  |  |  | 1 glass |  |  |  |
| 189 | Condensed milk/ RTD |  |  |  |  |  |  | 1 glass |  |  |  |
| 190 | Sugar |  |  |  |  |  |  | 1 glass |  |  |  |
| 191 | 3 in 1 |  |  |  |  |  |  | 1 sachet |  |  |  |
| **D** | **Fruit juice** | | | | | | | | | | |
| 192 | Fresh made |  |  |  |  |  |  | 1 glass |  |  |  |
| 193 | Commercial |  |  |  |  |  |  | 1 glass |  |  |  |
| 194 | Milk |  |  |  |  |  |  | 1 glass |  |  |  |
| **E** | **Other drinks** | | | | | | | | | | |
| 195 | Cordial/ Herbal drink (with added sugar) |  |  |  |  |  |  | 1 glass |  |  |  |
| 196 | Soy bean milk |  |  |  |  |  |  | 1 glass |  |  |  |
| 197 | Tetrapack drink (eg. sugar cane, chrysanthemum, lychee etc) |  |  |  |  |  |  | 1 packet |  |  |  |
| 198 | Carbonated/ soft drink |  |  |  |  |  |  | 1 can |  |  |  |
| **Sauces, Condiments & Soups** | | | | | | | | | | | |
| 199 | Budu/ cincaluk |  |  |  |  |  |  | 1 tbsp |  |  |  |
| 200 | Chilli sauce/ ketchup (Commercial) |  |  |  |  |  |  | 1 tbsp |  |  |  |
| 201 | Chutney - Santan |  |  |  |  |  |  | 1 tbsp |  |  |  |
| 202 | Chutney Non-santan |  |  |  |  |  |  | 1 tbsp |  |  |  |
| 203 | Mayonnaise |  |  |  |  |  |  | 1 tbsp |  |  |  |
| 204 | Rasam |  |  |  |  |  |  | 1 s.bowl |  |  |  |
| 205 | Sambal tumis |  |  |  |  |  |  | 1 tbsp |  |  |  |
| 206 | Sambal belacan |  |  |  |  |  |  | 1 tbsp |  |  |  |
| 207 | Soy sauce |  |  |  |  |  |  | 1 tbsp |  |  |  |
| 208 | Thousand island |  |  |  |  |  |  | 1 tbsp |  |  |  |
|  | Soup (only) | | | | | | | | | | |
| 209 | Clear (herbal) |  |  |  |  |  |  | 1 s.bowl |  |  |  |
| 210 | Cream (mushroom/ chicken) |  |  |  |  |  |  | 1 s.bowl |  |  |  |
| **Health supplements** | | | | | | | | | | | |
| 211 | Evening Primrose oil |  |  |  |  |  |  | 1 softgel |  |  |  |
| 212 | Fish oil |  |  |  |  |  |  | 1 softgel |  |  |  |
| 213 | Flaxseed oil |  |  |  |  |  |  | 1 tbsp |  |  |  |
| 214 | MCT, coconut oil |  |  |  |  |  |  | 1 tbsp |  |  |  |
| 215 | Olive oil |  |  |  |  |  |  | 1 tbsp |  |  |  |
| 216 | Sesame oil |  |  |  |  |  |  | 1 tbsp |  |  |  |
| 217 | Protein powder |  |  |  |  |  |  | 1 tbsp |  |  |  |
| **Finger foods**  *please specify the amount of curry or chilli sauce used | | | | | | | | | | | |
| 218 | Dim sum (pork)* |  | Fried |  |  |  |  | 1 piece |  |  |  |
| 219 | Dim sum (pork)* |  | Steamed |  |  |  |  | 1 piece |  |  |  |
| 220 | Dim sum (prawn)* |  | Fried |  |  |  |  | 1 piece |  |  |  |
| 221 | Dim sum (prawn)* |  | Steamed |  |  |  |  | 1 piece |  |  |  |
| 222 | Kebab/ Roti John (beef/chicken) |  |  |  |  |  |  | 1 set |  |  |  |
| 223 | Murtabak |  |  |  |  |  |  | 1 piece |  |  |  |
| 224 | Popiah (wet) |  |  |  |  |  |  | 1 piece |  |  |  |
| 225 | Popiah (fried) * |  |  |  |  |  |  | 1 piece |  |  |  |
| 226 | Sandwich, egg |  |  |  |  |  |  | 1 set |  |  |  |
| 227 | Sandwich, sardine/tuna |  |  |  |  |  |  | 1 set |  |  |  |
| 228 | Sushi* |  |  |  |  |  |  | 1 piece |  |  |  |
| 229 | Yong tauhoo (fried)* |  |  |  |  |  |  | 1 piece |  |  |  |
| 230 | Yong tauhoo (soup)* |  |  |  |  |  |  | 1 piece |  |  |  |
| 231 | Yong tauhoo (brinjal)* |  |  |  |  |  |  | 1 piece |  |  |  |
| **Fast food chain**  *please specify the amount of curry or chilli sauce used | | | | | | | | | | | |
| 232 | Fried Chicken *(KFC, McD etc)** |  |  |  |  |  |  | 1 piece |  |  |  |
| 233 | Hotdog, chicken/beef |  |  |  |  |  |  | 1 whole |  |  |  |
| 234 | French fries/ potato wedges* |  |  |  |  |  |  | 1 s.bowl |  |  |  |
| 235 | Coleslaw |  |  |  |  |  |  | 1 s.bowl |  |  |  |
| 236 | Pizza |  |  |  |  |  |  | 1 slice |  |  |  |
|  | Burger *(KFC, McD, Burger King, Ramly etc)* | | | | | | | | | | |
| 237 | Beef |  |  |  |  |  |  | 1 whole |  |  |  |
| 238 | Chicken |  |  |  |  |  |  | 1 whole |  |  |  |
| 239 | Fish |  |  |  |  |  |  | 1 whole |  |  |  |
| 240 | Banjo |  |  |  |  |  |  | 1 whole |  |  |  |
