# Supplementary material for: Providing Comprehensive Dietary Fatty Acid Profiling from Saturates to Polyunsaturates with the Malaysia Lipid Study-Food Frequency Questionnaire: Validation Using the Triads Approach
Source: Nutrients. 2020 Dec 31;13(1):120. doi: 10.3390/nu13010120 (PMC7823982; doi:10.3390/nu13010120)
Supplement: Supplementary file 1 [file nutrients-13-00120-s001.zip › nutrients-988928-Supplementary table.docx]

Table S1 Food item listing in the FFQ

| **No.** | **Food Groups** | **Subgroups** | **Total food items** |
| --- | --- | --- | --- |
| 1. | Cereals and products | 3 subgroups:  (a) biscuits  (b) bread  (c) others | 20 |
| 2. | Cooked rice | - | 11 |
| 3. | Noodles | 4 subgroups:  (a) soup-based  (b) non soup-based  (c) pasta  (d) others | 17 |
| 4. | Traditional *kuih* | 3 subgroups:  (a) Malay *kuih*  (b) Chinese *kuih*  (c) Indian *kuih* | 39 |
| 5. | Desserts, snacks and confectionary | - | 20 |
| 6. | Meat, poultry and products | - | 10 |
| 7. | Internal organs | - | 3 |
| 8. | Fish, shellfish and products | - | 11 |
| 9. | Legumes | - | 12 |
| 10. | Vegetables | - | 4 |
| 11. | Fruits and products | - | 18 |
| 12. | Milk and dairy products | - | 7 |
| 13. | Jam and spread | - | 7 |
| 14. | Beverages | 4 subgroups:  (a) alcohol  (b) coffee/ tea  (c) malted/ cereal/ chocolate drink  (d) fruit juice  (e) others | 19 |
| 15. | Sauces, condiments and soups | - | 12 |
| 16. | Health supplements | - | 7 |
| 17. | Finger foods | - | 14 |
| 18. | Fast food | - | 9 |

**Note:** *kuih*- sweet local cakes

**Table S2** Total energy, carbohydrate and protein by dietary assessment methods

| **Dietary Intake** | **DR** | | **FFQ** | | **Mean Difference, %** | ***P*-value*** |
| --- | --- | --- | --- | --- | --- | --- |
|  | **Mean ± SD** | **Median (IQR)** | **Mean ± SD** | **Median (IQR)** |  |  |
| Energy (kcal/day) | 1730±430 | 1683  (1434, 1958) | 1790±558 | 1664  (1384, 2092) | -3.5 | 0.410 |
| CHO (g/day) | 233.1±55.4 | 224.9  (197.8, 267.4) | 236.9±78.8 | 221.5  (183.0, 282.4) | -1.6 | 0.630 |
| Protein (g/day) | 65.0±21.6 | 60.9  (50.0, 76.7) | 68.4±25.5 | 64.4  (49.4, 81.5) | -5.2 | 0.290 |

Note- * significant if *P*<0.05

Abbreviations: DR=3-day dietary records; FFQ=food frequency questionnaires; g/day=gram per day; IQR- inter quartile range.

**Table S3** Correlations for total energy, carbohydrate and protein between FFQ and reference methods

| **Nutrients** | ***r*** | ***P*-value*** |
| --- | --- | --- |
| FFQ versus DR | | |
| Total Energy (kcal/day) | **0.441** | <0.001 |
| Carbohydrate (g/day) | **0.457** | <0.001 |
| Protein (g/day) | **0.329** | 0.001 |

Note- * significant if *P*<0.05

Abbreviations: DR=3-day dietary records; FFQ=food frequency questionnaires; g/day=gram per day.

**Table S4** Cross-quartile classifications for total energy, carbohydrate and protein between FFQ and reference methods

| **Nutrients** | **Same quartile (%)** | **Adjacent quartile (%)** | **Grossly misclassified (%)** |
| --- | --- | --- | --- |
| FFQ versus DR | | | |
| Total energy (kcal/day ^)^ | 38.9 | 38.9 | **5.6** |
| Carbohydrate (g/day) | 49.1 | 29.6 | **3.7** |
| Protein (g/day) | 32.4 | 39.8 | **4.6** |

Note: good agreement with <10% of gross misclassification was indicated by figures in bold.

Abbreviations: DR=3-day dietary records; FFQ=food frequency questionnaires; g/day=gram per day.
